# The NAC transcription factor ATAF2 enhances Arabidopsis biomass and chlorophyll *a* accumulations at the early growth stage

**DOI:** 10.1186/s13104-025-07421-x

**Published:** 2025-09-29

**Authors:** Hao Peng, Ying Zhai, Michael M. Neff

**Affiliations:** 1 USDA-ARS Crop Diseases, Pests and Genetics Research Unit, Parlier, CA 93648 USA; 2 Department of Crop and Soil Sciences, Washington State University, Pullman, WA 99164 USA

**Keywords:** *Arabidopsisthaliana*, ATAF2, Auxin, Biomass, Brassinosteroids, Chlorophyll, NYC1

## Abstract

**Objective:**

The *Arabidopsis thaliana* NAC-family transcription factor ATAF2 plays extensive regulatory roles in plant disease resistance, abiotic stress tolerance, leaf senescence, hormone metabolism, and seedling photomorphogenesis. Using Arabidopsis seedlings as the investigation platform, we previously demonstrated that *ATAF2* overexpression can increase the endogenous levels of the growth-promoting hormone brassinosteroids (BRs) and suppress the expression of the chlorophyll *b* (Chl-*b*) reductase NYC1, which catalyzes the initial step of the degradation of light-harvesting chlorophyll *a*/*b*-protein complex of photosystem II (LHCII). ATAF2 also promotes the expression of *NIT2*, which is involved in the biosynthesis of the auxin indole-3-acetic acid (IAA). Here, we further examined the effects of elevated BR/IAA levels and reduced NYC1 expression on biomass and Chl-*a*/*b* accumulations, respectively.

**Results:**

Twelve-day-old plants were harvested for biomass and Chl-*a*/*b* measurements. While no significant difference of biomass or Chl-*a*/*b* accumulations was observed between the wild-type Col-0 and the loss-of-function *ataf2-1/2* plants, all three *ATAF2* overexpression lines (*ATAF2ox-1/2/3*) exhibited much higher biomass and Chl-*a* accumulations as compared to Col-0 and *ataf2-1/2*, which can at least be partially interpreted as the consequences of higher endogenous BR/IAA levels and reduced *NYC1* expression, respectively. The results demonstrate the positive regulatory role of ATAF2 in biomass and Chl-*a* accumulations. Notably, *ATAF2* overexpression does not increase Arabidopsis biomass accumulation at later growth stages, indicating its functional nature of developmental timing acceleration.

## Introduction

The NAM/ATAF/CUC (NAC)-family proteins are a large group of plant-specific transcription factors (TFs) functioning in biotic/abiotic stress responses and hormonal/developmental regulations [1]. *Arabidopsis thaliana* ATAF-group NAC TF ATAF2 (ANAC081, AT5G08790) plays extensive transcriptional activation and/or repression [2] roles in fungal [3] and viral [4, 5] resistance, auxin [6] and ethylene [7] biosynthesis and signaling [8], brassinosteroid (BR) inactivation [9], seedling photomorphogenesis [9, 10], abscisic acid (ABA)- [11] and dark-induced [12, 13] leaf senescence, and abiotic stress tolerance [7, 8]. ATAF2 specifically binds to the EE/CBS circadian elements [9] and other A/T-rich promoter motifs [14], and fulfills its functions via physical and/or genetic interactions with multiple partner proteins, including its two closest homologs ANAC102 and ATAF1 (ANAC002) [11, 15], additional stress-responsive NAC TFs ANAC019/032/055/072 [11], the retrograde ethylene and auxin signaling regulator ANAC017 [8], the NAC TF VNI2 (ANAC083) that negatively modulates xylem vessel formation [13], other downstream leaf-senescence-regulating NAC TFs ANAC029/042/046/059/092 [12], two AT-hook-binding proteins AHL12/27 that modulate light-responsive hypocotyl growth inhibition [16], the primary far-red photoreceptor phytochrome A (phyA) [9, 10], and the core circadian oscillation regulator CCA1 [15, 17]. ATAF2 also binds to the promoter of *NIT2*, which is involved in the biosynthesis of the auxin indole-3-acetic acid (IAA) [6]. The binding of ATAF2 significantly promotes *NIT2* expression, which is confirmed by both RNA blot/RT-qPCR [6] and our RNA-seq-based transcriptome [7] assays.

We previously reported that ATAF2 directly binds both EE and CBS *cis*-elements in the promoters of two BR-inactivating cytochrome P450 (CYP) genes *BAS1* (*CYP734A1*, formerly *CYP72B1*, AT2G26710) [18] and SOB7 (*CYP72C1*, AT1G17060) [19] to suppress their expression [9]. Brassinolide (BL) and castasterone (CS) are two end products of the BR biosynthetic pathway [18, 19]. They are also the two BR species exhibiting the strongest biological activities. BRs are well known to promote cell elongation and plant growth [20, 21]. Consistently, in response to dim white light or exogenous BR treatments, *ATAF2* gain- and loss-of-function mutants exhibit long- and short-hypocotyl phenotypes, respectively [9]. Transcriptome analysis was performed using Arabidopsis seedlings of the wild-type Columbia (Col-0) ecotype, two T-DNA insertional knockout mutants *ataf2-1* (SALK_136355C) and *ataf2-2* (SALK_015750C), and three *ATAF2* overexpression lines *ATAF2ox-1/2/3* [7]. Significantly decreased transcript accumulation of *NON-YELLOW COLORING 1 (NYC1)* was observed in all three *ATAF2ox* lines, which was further verified by RT-qPCR [7]. NYC1 encodes a chlorophyll *b* (Chl-*b*) reductase involved in the initial step of the degradation of light-harvesting chlorophyll *a*/*b*-protein complex of photosystem II (LHCII) [22–24].

In this extended research built upon our previous work on ATAF2 functions [7, 9], we measured the biomass accumulation and Chl-*a*/*b* concentrations in Col-0, *ataf2-1/2*, and *ATAF2ox-1/2/3* mutants at the post-seedling stage (12 days after germination). The results experimentally confirmed that compared to *ATAF2* loss-of-function mutants (*ataf2-1/2*) and the wild type (Col-0), *ATAF2* gain-of-function plants (*ATAF2ox-1/2/3*) exhibited significantly larger weights and much higher Chl-*a* accumulations, which can at least be partially interpreted as the consequences of higher endogenous BR/IAA levels and reduced *NYC1* expression, respectively. Notably, Chl-*b* accumulations did not show significant variation across all six genotypes. Increased Chl-*a* accumulation may help stabilize the photosynthetic apparatus LHCII, which can also contribute to biomass production together with BRs and IAA. However, *ATAF2* overexpression does not increase Arabidopsis biomass accumulation at later growth stages, indicating its functional nature of developmental timing acceleration.

## Materials and methods

### Plant materials and growth conditions

All Arabidopsis germplasms used in this research are derived from the Col-0 ecotype. T-DNA insertional mutants *ataf2-1* and *ataf2-2* were obtained from the Arabidopsis Biological Resource Center (ABRC). *ATAF2ox-1*, *ATAF2ox-2*, and *ATAF2ox-3* are three independent and homozygous single-T-DNA-insertion lines that overexpress ATAF2 using the CaMV 35S promoter. They have been described in our previous reports [7, 9]. Knockout or overexpression of ATAF2 in these lines was confirmed by both RT-qPCR and RNA-seq [7, 9]. The wild-type Col-0, *ataf2-1/2*, and *ATAF2ox-1/2/3* seeds were surface-sterilized for 20 min using a shaker and 70% ethanol solution supplemented with 0.01% Tween 20. Seeds were then washed with 95% ethanol for 20 min on a shaker, spread and air-dried on sterilized filter papers in a laminar flow hood, and transferred to half-strength (1/2) Murashige and Skoog medium (MS) [25] agar plates using sterilized toothpicks. 1/2 MS petri dishes with seeds on their surfaces were sealed with 3 M micropore tapes, wrapped in aluminum foil, and put in darkness at 4 °C for 4 days. After cold stratification, plates were incubated at 25 °C under continuous white light in a plant growth chamber (Percival Scientific E-30L1, Perry, IA, USA). Five days after germination, uniformly grown seedlings of each genotype were transferred to soil and cultured for one more week. Compared to direct seed sewing in soil, the use of plant growth medium can promote homogeneous seed germination and seedling growth [9], and thereby improving the reproducibility of subsequent assays.

### Measurements of plant fresh/dry weight, chlorophyll concentration, Rosetta leaf number and chlorophyll fluorescence

After counting Rosetta leaf numbers, 12-day-old (12 d) intact plants were pulled out of soil, cleaned up soil debris in water, and dried on paper towels before weighing on a high-precision analytical balance (Mettler-Toledo AB104-S, Columbus, OH, USA). Chlorophyll extraction and concentration estimation were performed as described previously [26]. After weighing, each plant was put into a 1.5-mL Eppendorf tube prefilled with 1 mL of 80% acetone solution and shaken overnight at 200 RPM and room temperature. After centrifugation for 5 min at 15,000 g, the supernatants were used for absorbance (*A*) measurements at wavelengths 646 (*A*_646_) and 663 (*A*_663_) nm. The measurements were carried out using transparent 96-well ELISA plates (200-μL supernatant per sample per well) and a Spark multimode microplate reader (Tecan Life Sciences, Männedorf, Switzerland). Chl-*a*, Chl-*b*, and total Chl concentrations were estimated using the Lichtenthaler’s equations [27] as follows: Chl-*a* (μg/mL) =  − 1.93*A*_646_ + 11.93*A*_663_; Chl-*b* (μg/mL) = 20.36*A*_646_ − 5.50*A*_663_; Total Chl (μg/mL) = 6.43*A*_663_ + 18.43*A*_646_. All Chl concentration values were then converted to the format of Chl-*a*, Chl-*b*, and total Chl content (μg) per gram of fresh plant weight (μg/g). Thirty biological replicates of plants from each genotype were used for fresh weight and subsequent chlorophyll concentration measurements.

Additional plants were used for measurements of 27 and 37 d old plant fresh weight, as well as 12, 27, and 37 d old plant dry weight. Since plants were grown in continuous light with no dark adaptation period, we did not measure relevant leaf chlorophyll fluorescence parameters such as dark variable (*Fv*) and maximum (*Fm*) fluorescence, quantum efficiency of photosystem II (PSII) in the dark (*Fv*/*Fm*), or non-photochemical quenching (NPQ) [28]. Instead, a LI-600 porometer/fluorometer (LI-COR Environmental, Lincoln, NE, USA) was used to measure light minimum (*Fs*) and maximum (*Fm*ʹ) fluorescence, PSII quantum efficiency (ΦPSII) calculated by (*Fm*ʹ−*Fs*)/*Fm*ʹ, and electron transport rate (ETR; μmol electrons m^−2^ s^−1^) of 20 days old Rosetta leaves.

For all the measurement results of the six plant genotypes, the significance of differences was determined by one-way ANOVA with Tukey’s honestly significant difference (HSD) test. Groups with significant differences (*P* < 0.05) are labeled by different letters.

## Results and discussion

### *ATAF2* overexpression increases biomass accumulation in Arabidopsis seedlings

**Fig. 1:**
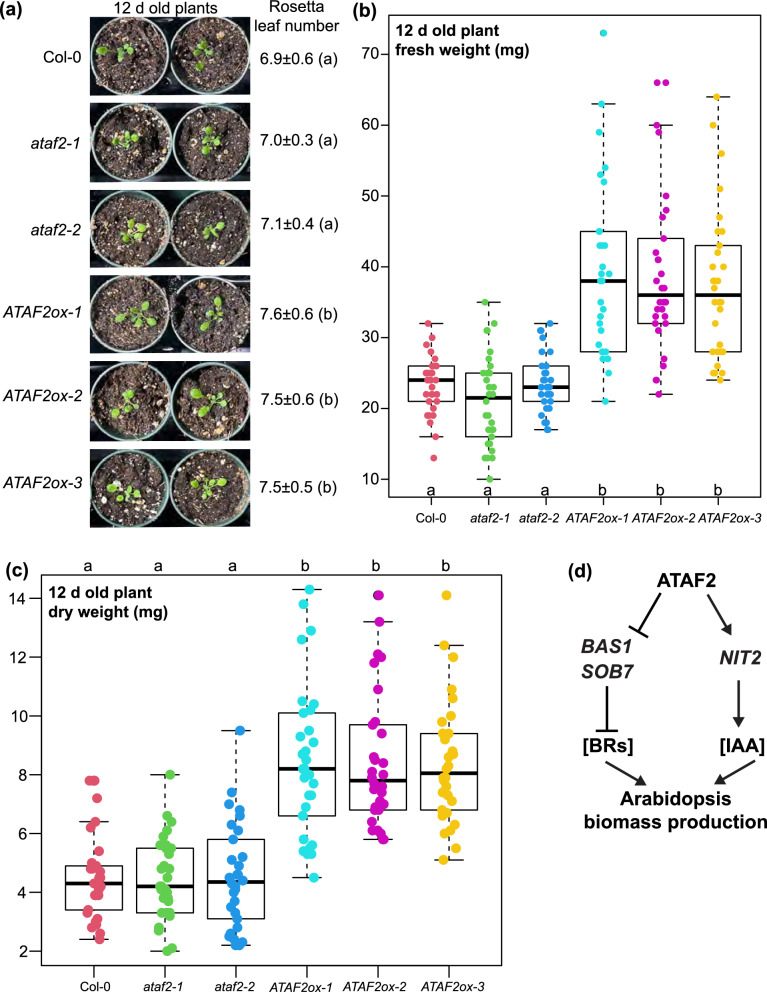
*ATAF2* overexpression leads to significantly higher biomass accumulation in Arabidopsis. **a** 12 d old wild-type (Col-0), T-DNA insertional knockout mutants (*ataf2-1* and *ataf2-2*), and *ATAF2* overexpression plants (*ATAF2ox-1*, *ATAF2ox-2*, and *ATAF2ox-3*) were used for Rosette leaf number counts (number ± SD) and subsequently harvested for fresh and dry weight measurements. All three *ATAF2* overexpression lines showed significantly higher Rosetta leaf numbers as compared to Col-0 and the two *ATAF2* knockout mutants. **b**, **c** Compared to Col-0 and *ataf2-1/2*, *ATAF2ox-1/2/3* plants exhibited much higher biomass accumulation as reflected by their fresh and dry weights. Rosette leaf numbers (**a**), fresh (**b**) and dry (**c**) weights of 30 biological replicates of plants (*n* = 30) were measured for each genotype. The significance of differences was determined by one-way ANOVA with Tukey’s HSD test. Groups with significant differences (*P* < 0.05) are labeled by different letters. **d** A model dissecting the underlying mechanisms of ATAF2-enhanced biomass production. ATAF2 suppresses the expression of BR-inactivating genes *BAS1* and *SOB7*. Reduced production of BAS1 and SOB7 enzymes leads to elevated endogenous BR levels in Arabidopsis. ATAF2 also promotes the expression of IAA biosynthetic gene *NIT2*, which increases endogenous IAA level. Higher BR and IAA levels result in more biomass accumulation

12 d old plants were harvested for fresh weight measurements (Fig. 1a). The petiole lengths of *ataf2-1* and *ataf2-2* were notably shorter than those of Col-0 and *ATAF2ox-1/2/3* (Fig. 1a). Compared to Col-0 and the two *ATAF2* knockout lines *ataf2-1/2*, all three *ATAF2* overexpression lines (*ATAF2ox-1/2/3*) had slightly but significantly higher Rosetta leaf number counts (Fig. 1a). Col-0 and *ataf2-1/2* showed similar biomass accumulation rates, with average fresh weights ranging around 21 to 23 mg (Fig. 1b). In contrast, *ATAF2ox-1/2/3* exhibited much higher biomass accumulation, with 37 to 39 mg of fresh weights on average (Fig. 1b). Consistently, *ATAF2* overexpression also led to significantly higher dry weight in Arabidopsis (Fig. 1c).

BRs [29, 30], auxins [29, 31], and ethylene [31, 32] are all positive regulators of petiole growth and elongation. Disruption of *ATAF2* causes reductions of endogenous BR [9], IAA [6], and ethylene [7] levels in Arabidopsis, which may account for the short-petiole phenotype in *ataf2-1* and *ataf2-2* (Fig. 1a). In addition, both BRs [33–35] and auxins [36–38] promote plant biomass production at reasonable concentrations. Changes of hormone homeostasis can also be used to develop a model addressing the increase of biomass accumulation in *ATAF2* overexpression plants (Fig. 1d). In the first pathway of this model, ATAF2 suppresses the expression of two BR-inactivating CYP genes *BAS1* and *SOB7* [9]. Both BAS1 and SOB7 reduce plant endogenous BR levels [18, 19]. *ATAF2* overexpression leads to increased endogenous BR levels and results in higher biomass accumulation (Fig. 1d). In the second pathway of this model, ATAF2 activates *NIT2* expression [6]. NIT2 promotes IAA production [39, 40]. Increased IAA level caused by *ATAF2* overexpression enhances biomass accumulation (Fig. 1d).

Since NIT1 rather than NIT2 is the major nitrilase responsible for indole-3-acetonitrile (IAN) conversion to IAA [41] in Arabidopsis, the ATAF2-NIT2-IAA pathway may not be as important as the ATAF2-BAS1/SOB7-BR module in biomass production. On the other hand, ethylene generally inhibits plant vegetative growth and biomass accumulation [42, 43]. Thus, ATAF2-induced 1-aminocyclopropane-1-carboxylic acid (ACC) synthase (ACS) 4/5 expression and ethylene overproduction [7] may antagonize the biomass accumulation promoted by IAA and BRs. The overall net outcome of ATAF2-regulated hormone homeostasis still leans towards the increase of biomass production.

### *ATAF2 *overexpression increases Chlorophyll *a* accumulation in Arabidopsis seedlings

**Fig. 2:**
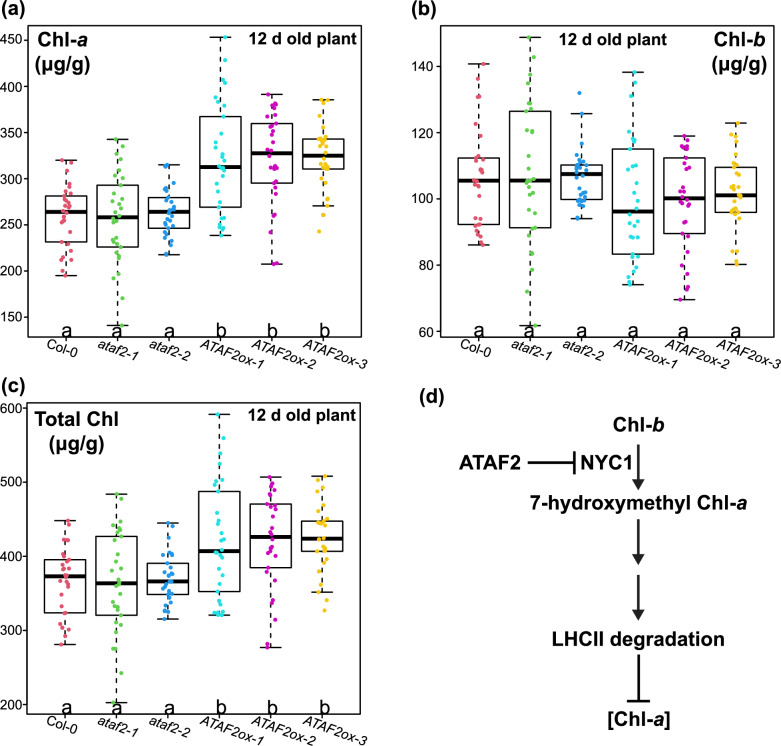
*ATAF2* overexpression leads to significantly higher chlorophyll *a* (Chl-*a*) accumulation in Arabidopsis. **a**–**c** 12d old plants were used for total Chl extraction and Chl-*a*/*b* estimation immediately after fresh weight measurements. Chl-*a*, Chl-*b*, and total Chl contents (μg) per gram of fresh plant weight (μg/g) were then calculated. **a** Compared to the wild-type (Col-0) plants and T-DNA insertional knockout mutants (*ataf2-1* and *ataf2-2*), all three *ATAF2* overexpression lines (*ATAF2ox-1*, *ATAF2ox-2*, and *ATAF2ox-3*) exhibited significantly higher Chl-*a* accumulation. **b** In contrast, Chl-*b* contents showed no significant difference across all six genotypes. **c** *ATAF2ox-1*/*2*/*3* also exhibited significantly higher total Chl accumulation, attributed to higher accumulation of Chl-*a* in these plants. Chl-*a*, Chl-*b* and total Chl concentrations of 30 biological replicates of plants (*n* = 30) were measured for each genotype. The significance of differences was determined by one-way ANOVA with Tukey’s HSD test. Groups with significant differences (*P* < 0.05) are labeled by different letters. **d** A model dissecting the underlying mechanism of ATAF2-enhanced Chl-*a* accumulation. ATAF2 suppresses the expression of *NYC1*, which encodes a Chl-*b* reductase. NYC1 converts Chl-*b* to 7-hydroxymethyl Chl-*a* (HMChl) and is involved in subsequent LHCII degradation. Reduced NYC1 supply caused by *ATAF2* overexpression may attenuate LHCII degradation and thereby facilitating Chl-*a* accumulation

The average Chl-*a* concentrations in 12 d old Col-0, *ataf2-1*, and *ataf2-2* plants were all around 260 μg/g of fresh plant weight (Fig. 2a). In contrast, all *ATAF2ox-1*, *ATAF2ox-2*, and *ATAF2ox-3* plants showed much higher Chl-*a* content, averaging at about 320 μg/g (Fig. 2a). However, plants from all six genotypes possessed similar concentrations of Chl-*b* (about 100 to 107 μg/g on average), with no statistically significant difference determined by one-way ANOVA (Fig. 2b). Owing to elevated Chl-*a* levels, all three *ATAF2* overexpression lines had significantly higher concentrations of total Chl (about 420 to 425 μg/g on average) as compared to Col-0 and the two ATAF2 knockout mutants (about 365 to 370 μg/g of total Chl on average) (Fig. 2c).

The supply of the Chl-*b* reductase NYC1 plays a central role in our mechanistic model interpretating ATAF2-enhanced Chl-*a* accumulation (Fig. 2d). *ATAF2* overexpression reduces the transcript accumulation of *NYC1* [7], leading to lower availability of the Chl-*b* reductase. Since NYC1 converts Chl-*b* to 7-hydroxymethyl Chl-*a* (HMChl) and causes subsequent LHCII degradation [22], reduced amount of NYC1 can stabilize LHCII and its Chl-*a*/*b* components, resulting in higher Chl-*a* accumulation (Fig. 2d). Notably, 7-hydroxymethyl chlorophyll *a* reductase (HCAR) can convert HMChl generated by NYC1 to Chl-*a* in the chlorophyll cycle [44]. Thus, ATAF2-mediated repression of *NYC1* expression may reduce the amount of Chl-*a* converted from HMChl. Overall, ATAF2-induced Chl-*a* stabilization in LHCII complex outperforms the loss from reduced HMChl-to-Chl-*a* conversion, resulting in net Chl-*a* accumulation.

ATAF2-mediated *NYC1* suppression can increase Chl-*b* accumulation by reducing Chl-*b*-to-HMChl conversion and stabilizing Chl-*b* in LHCII. However, we did not see significant change of Chl-*b* accumulation in *ATAF2* overexpression lines (Fig. 2b). The modulation of multiple metabolic and signaling pathways may contribute to achieving Chl-*b* homeostasis, including the chlorophyllide *a* oxygenase (CAO) responsible for Chl-*b* biosynthesis [45], STAY-GREEN (SGR) proteins involved in Chl *a*/*b* degradation [46], the feedback network regulation of the chlorophyll cycle enzymes [23], BALANCE of CHLOROPHYLL METABOLISM (BCM) proteins modulating the trade-off between Chl synthesis and breakdown [47], and additional regulators of Chl degradation such as another Arabidopsis NAC TF ANAC046 [48].

### *ATAF2* overexpression does not increase Arabidopsis biomass accumulation at later growth stages

**Fig. 3:**
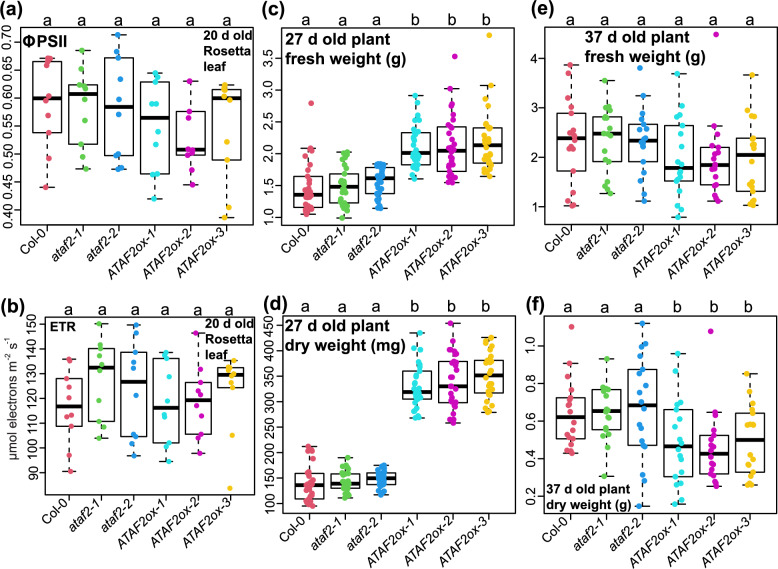
*ATAF2* overexpression does not increase Arabidopsis biomass accumulation at later growth stages. 20 d old Col-0, *ATAF2* knockout and overexpression plants all exhibited similar levels of ΦPSII (**a**) and ETR (**b**) values. 27 d old *ATAF2* overexpression plants still showed significantly higher fresh (**c**) and dry (**d**) weights as compared to Col-0 and *ATAF2* knockout lines. (**e**) All six genotypes of 37 d old plants had similar fresh weights. (**f**) The *ATAF2* overexpression lines showed significantly lower dry weights as compared to Col-0 and *ATAF2* knockout lines. Ten biological replicates of Rosetta leaves (*n* = 10) from each genotype were used for ΦPSII and ETR measurements. Thirty biological replicates of 27 d old plants (*n* = 30) from each genotype were used for fresh and dry weight measurements. Eighteen biological replicates of 37 d old plants (*n* = 18) from each genotype were used for fresh and dry weight measurements. The significance of differences was determined by one-way ANOVA with Tukey’s HSD test. Groups with significant differences (*P* < 0.05) are labeled by different letters

When Arabidopsis Rosetta leaves grew large enough for fluorescence detections on day 20, we measured ΦPSII (Fig. 3a) and ETR (Fig. 3b) of all six genotypes. Unlike younger seedlings, 20 days old plants of Col-0, *ATAF2* knockout and overexpression lines all exhibited similar levels of ΦPSII and ETR values, indicating that ATAF2 may no longer contribute to LHCII stabilization when plants grow older. Largely due to faster growth and biomass accumulation at the seedling stage, 27 days old *ATAF2* overexpression plants still showed significantly higher fresh (Fig. 3c) and dry (Fig. 3d) weights as compared to Col-0 and *ATAF2* knockout lines. However, all six genotypes of 37 days old plants had similar fresh weights (Fig. 3e). The *ATAF2* overexpression lines even showed significantly lower dry weights (Fig. 3f), probably due to their earlier-than-normal senescence [12] and cease of growth.

## Conclusion

The NAC TF ATAF2 modulates BR catabolic and IAA biosynthetic pathways to elevate endogenous BR/IAA levels and promote biomass accumulation in Arabidopsis. ATAF2 suppresses *NYC1* expression to boost Chl-*a* accumulation. Increased Chl-*a* accumulation may improve LHCII stability, thereby enhancing photosynthesis and contributing to biomass production. Changes of biomass and Chl-*a* accumulations are only observed in *ATAF2* overexpression lines, which may be due to functional redundancy of ATAF2 and its homologous NACs [11, 15]. Although ATAF2 promotes Arabidopsis seedling growth, it does not enhance biomass or Chl-*a* accumulation at later growth stages, suggesting that ATAF2 may serve as a developmental timing accelerator rather than a sustainable biomass accumulation enhancer throughout the plant life cycle.

## Limitations

The relative contributions of BRs and IAA to ATAF2-mediated biomass accumulation in Arabidopsis seedlings are still unclear and yet to be clarified. One future direction would be to cross an *ATAF2* overexpression line into auxin- or BR-deficient mutant backgrounds and then examine the biomass accumulation phenotype. This kind of genetic analysis would provide a more mechanistic understanding of how ATAF2 influences plant seedling growth.

ATAF2 only promotes Arabidopsis biomass and Chl-*a* accumulations at early growth stages. It is much easier to establish uniform growth conditions for small-size plants (12 days after germination), and more convenient to separate plants from soil and measure Chl *a*/*b* contents at this stage. The data generated can be more accurate and comparable. It should be aware that the expression of multiple leaf senescence-associated *NAC* genes are not regulated by ATAF2 at the seedling stage [7]. However, ATAF2 can activate the expression of these *NAC*s, promote leaf senescence, and induce Chl breakdown when plants get older [12]. It is likely that ATAF2 initially promotes plant growth and Chl-*a* accumulation, but induces leaf senescence and Chl degradation later on. This hypothesis is supported by our observations that ATAF2 failed to improve biomass accumulation, leaf ΦPSII or ETR at later growth stages.

## Data Availability

Data is provided within the manuscript. The datasets used and/or analyzed during the current study are available from the corresponding author on reasonable request.

## Abbreviations

*A*: Absorbance
ABA: Abscisic acid
ACC: 1-Aminocyclopropane-1-carboxylic acid
ACS: ACC synthase
ABRC: Arabidopsis Biological Resource Center
AHL: AT-HOOK MOTIF NUCLEAR LOCALIZED
ANOVA: Analysis of variance
ARS: Agricultural Research Service
ATAF: *Arabidopsis thaliana* Activating Factor
BCM: BALANCE of CHLOROPHYLL METABOLISM
BL: Brassinolide
BR: Brassinosteroid
CAO: Chlorophyllide *a* oxygenase
CBS: CCA1-Binding Site
CCA1: CIRCADIAN CLOCK ASSOCIATED 1
Chl-*a*: Chlorophyll *a*
Chl-*b*: Chlorophyll *b*
Col-0: Columbia
CS: Castasterone
CUC: CUP-SHAPED COTYLEDON
CYP: Cytochrome P450
ECDRE: Emergency Citrus Disease Research and Extension Program
EE: Evening Element
ELISA: Enzyme-linked immunosorbent assay
ETR: Electron transport rate
*Fm*: Maximum fluorescence in the dark
*Fm'*: Maximum fluorescence in the light
*Fs*: Minimum fluorescence in the light
*Fv*: Variable fluorescence in the dark
*Fv/Fm*: Quantum efficiency of photosystem II in the dark
*g*: Relative centrifugal force (RCF)
HCAR: 7-Hydroxymethyl chlorophyll *a* reductase
HMChl: 7-Hydroxymethyl chlorophyll *a*
HSD: Honestly significant difference
IAA: Indole-3-acetic acid
IAN: Indole-3-acetonitrile
LHCII: Light-harvesting chlorophyll *a*/*b*-protein complex of photosystem II
MS: Murashige and Skoog medium
NAC: NAM/ATAF/CUC
NAM: NO APICAL MERISTEM
NIFA: National Institute of Food and Agriculture
NIT1: Nitrilase 1
NIT2: Nitrilase 2
NPQ: Non-photochemical quenching
NYC1: NON-YELLOW COLORING 1
RNA-seq: RNA sequencing
RPM: Revolutions per minute
RT-qPCR: Reverse transcription quantitative PCR
SD: Standard deviation
SGR: STAY-GREEN
TF: Transcription factor
USDA: United States Department of Agriculture
VNI2: VND-INTERACTING 2
ΦPSII: Quantum efficiency of photosystem II in the light, calculated by (*Fm*ʹ-*Fs*)/*Fm*
